# Maximizing micro-grid energy output with modified chaos grasshopper algorithms

**DOI:** 10.1016/j.heliyon.2024.e23980

**Published:** 2024-01-03

**Authors:** Zhiyu Yan, Yimeng Li, Mahdiyeh Eslami

**Affiliations:** a College of Electrical Engineering, Yellow River Conservancy Technical Institute, Kaifeng 475004, Henan, China; b Department of Electrical Engineering, Skills Training Center of the State Grid Jibei Electric Power Company Limited (Baoding Electric Power Vocational and Technical College), Baoding 071051, Hebei, China; c Department of Electrical Engineering, Kerman Branch, Islamic Azad University, Kerman, Iran; d College of Technical Engineering, The Islamic University, Najaf, Iraq

**Keywords:** Modified chaos grasshopper algorithm (MCGA), Techno-economic energy management strategy (TEMS), Photovoltaic, Fuel cell, Daily electricity price

## Abstract

This study presents a Modified version of Chaos Grasshopper Algorithm (MCGA) as a solution to the Techno-Economic Energy Management Strategy (TEMS) problem in microgrids. Our main contribution is the optimization of parameters to minimize the overall daily electricity price in an integrated clean energy micro-grid, incorporating fuel cell, battery storage, and photovoltaic systems. Through comparative simulations with established methods (HOMER, GAMS, GWO, and MILPA), we demonstrate the superiority of our proposed strategy. The results reveal that MCGA surpasses these methods, yielding significantly improved optimal solutions for the overall daily electricity price. Notably, the MCGA approach exhibits high precision, flexibility, and adaptability to power prices and environmental constraints, leading to accurate and flexible solutions. Thus, our proposed approach offers a promising and effective solution for the TEMS problem in microgrids, with the potential to greatly enhance microgrid performance.

## Nomenclature

$L_{PV}$: The solar panel loss factor
$\beta_{TP}$: The power temperature coefficient
$P_{PV}^{POR}$: The output power ratio capacity for the PV panel
$n_{\text{PV}}$: The PV panel ratio
$\bar{T}$: The pv temperature under standard test conditions
$G_{s}$: The standard incident radiation ($1000W/m^{2}$)
$G_{h}\left(t\right)$ r: The hourly pv panel solar radiation incident
$T_{c}$: The solar cell temperature
$T_{A}$: The ambient temperature
$V_{act}$: The activation voltage loss
$V_{\omega }$: The ohmic voltage loss
$V_{cons}$: The concentration voltage loss
$E_{N}$: The Nernst reversible voltage
R: The gas universal constant equal to $8.314\;kJ\;{\left(kmol\;K\right)}^{-1}$
$\varphi_{Bat}$: The phase-shift
$E_{B}$: The dc bus accessible energy
$P_{G}$: The generated energy for the grid
$P_{L}$: The generated energy for the load
$P_{PV}$: The generated energy for the photovoltaic
$P_{bat}$: The generated energy for the battery
${\dot{E}}_{B}^{r}$: The required power of the common bus
$W_{i\;}$: The wind advection
$S_{i\;}$: The social contact
T: The fuel cell's operating temperature
F: The Faraday constant which equal to $96,486\;C\;mol^{-1}$
$E_{0}$: The standard potential
$P_{H_{2}}$: The hydrogen pressures
$P_{O_{2}}$: Oxygen pressures
$P_{H_{2}O}$: Water partial pressures
$N_{cell}$: The number of the combined cells
A: The available area
$V_{b}$: Voltage source
$R_{b}$: Internal resistance
$I_{B}$: The battery current
$E_{B}$: The potential of the battery
$N_{B}$: The lithium quantity
$R_{B}$: The real part of the resistance
$C_{B}$: The capacity
$V_{B}$: Voltage bus
$E_{P}$: Electricity pricing
N: The quantity of the power sources
EP(t): The pricing of the electricity market
$SoC^{*}\left(t\right)$: The optimal rate of the soc
$\omega_{n}$: The cutoff frequency
ζ: Damping ratio
$G_{i\;}$: The gravity force

## 1 Introduction

Rapid population growth, urbanization, and industry are the main causes of the rising energy demand [1]. Since they are not only sustainable but also economical, alternative energy sources are gaining popularity as a result of the substantial financial savings they may produce. Between 2020 and 2040, it is predicted that the world's energy consumption will rise by 37 %, necessitating the development of new energy sources [2]. Providing us with power, heating, cooling, and other services, energy is a crucial component of contemporary life [3].

Although fossil fuels are the most widely utilized energy source, they are non-renewable and have a limited supply [4]. This has prompted a quest for alternative energy sources that are renewable and sustainable. As a green response to the expanding energy demand, these resources have grown in prominence over the past few centuries [5].

As the globe strives for cleaner and more effective sources of electricity, alternative energy sources including solar, wind, hydropower, geothermal, biomass, and nuclear power are growing more and more significant [6]. These forms of energy can lessen pollution and lessen the consequences of worldwide warming and are less expensive than fossil fuels [7]. Utilizing these new energy sources and using energy more effectively is key to ensuring the future supply of energy [8].

Today, the globe relies heavily on electricity to fulfill a variety of demands. The problems with the current power management systems get worse as long as there is a continued increase in the electricity demand. Microgrids are crucial for electricity systems in this regard [9].

Traditional power systems are under tremendous strain as a result of the rising electricity demand, which might result in regular outages or blackouts. Microgrids are a good option for managing the power supply more effectively [10]. Small electrical systems known as microgrids are generally linked to the primary utility grid but may be cut off from it under specific circumstances, such as a grid breakdown or an emergency [11]. The microgrid can build an "islanded" network of users and can offer electricity backup electricity [12].

The term "microgrid" refers to a small-scale network of distributed resources for energy that are often situated near other resources. It is made up of several parts, including local loads, energy storage systems, solar panels, wind farms, managing and controlling networks, and technology for communications [13]. The desire to lower greenhouse gas emissions and improve the reliability and safety of the electrical supply is what is driving the growth of microgrids. Microgrids provide a higher level of autonomy, enabling users to control their energy generation and consumption, which can be advantageous for the energy sector's financial and environmental impacts [14].

By utilizing renewable energy sources, lowering greenhouse gas emissions, and promoting green energy generation and consumption, microgrids may support an energy system that is favorable to the environment.

Management of electricity is becoming increasingly significant in today's society. Due to their capacity to deliver safe, dependable, and affordable energy services, microgrids have shown to be a successful option. An overview of microgrids and their significance as a crucial element of the ideal energy management system is given in this study [15]. Organizations may improve energy effectiveness and financial savings, lessen dependency on the main electrical system, and deliver electricity to remote regions or those with restricted access to the main electrical network by utilizing the special characteristics of microgrids [16].

Any ideal energy management system must include microgrids for enterprises to achieve the highest levels of energy efficiency and cost savings. Microgrids will be even more crucial as a tool for guaranteeing efficient and effective energy management as technology develops [17]. AI is being utilized to address complicated challenges in microgrids, and managing electricity optimization is becoming more and more crucial to how microgrids are run. The advantages of employing AI to solve these problems will be covered in this article along with any potential obstacles that may need to be solved to fully realize AI's capabilities. The article will focus on the role of AI in addressing microgrids by applying energy management optimization methodologies and technologies [18].

Energy management optimization is the process of adjusting a system's energy usage depending on its present and potential future energy needs, such as a microgrid. AI assistance or physical labor are also options for completing this. Reduced energy use, financial savings, and a sustainable energy supply are the goals of energy management optimization [19]. To optimize energy management, AI is mostly used to identify effective solutions to challenging issues that arise inside the microgrid. Artificial intelligence (AI) algorithms can quickly evaluate vast volumes of data to find patterns and trends. These discoveries may then be applied to optimize the microgrid's energy use and raise its effectiveness [20].

The use of AI to optimize energy management has several advantages [21]. AI algorithms, for instance, may be used to automate energy management choices and more accurately estimate energy consumption. AI-based solutions can be employed as well to find potential for energy savings and offer suggestions for how to improve energy use. AI may also facilitate more efficient processes for making decisions and lower operational expenses [22].

An effective method for raising the effectiveness and sustainability of microgrids is energy management optimization. Engineers and scientists can handle challenging issues in the microgrid by using AI for energy management optimization. Utilizing AI to optimize energy management has numerous potential advantages, but certain hazards and difficulties need to be taken into consideration [17].

Energy management is now more crucial than ever as the globe enters a new era of technological development [22]. Systems for managing energy have been created to maximize the use of existing energy resources as many nations work to make their networks "greener" and minimize emissions [23]. To enhance the efficiency and dependability of the system, these systems employ optimization approaches to utilize and integrate alternative energy sources, such as solar and wind power. This requires applying several iterations of an optimization method to the system [24].

Technology improvements have made it possible for researchers to identify methods to enhance managing electricity optimization in microgrids as the globe faces more complicated energy concerns. Small-scale power systems known as microgrids are used to supply electricity to nearby areas. They are often linked to the primary electrical grid [25]. They are playing a bigger role in the effective and trustworthy distribution of energy, especially in places with few resources and poor access to power. Since various studies on the improvement of energy management in microgrids have been carried out recently and are discussed in the following examples, there has been an increase in interest in the study and development of energy management systems for microgrids.

M. A Kamarposhti [26] and colleagues proposed a management system for optimal microgrid operations, factoring in existing capacities in the electricity market. The operator of the microgrid, responsible for its secure operations, must engage in a planning process that makes the most of the network's components. To achieve this, they sought to guarantee adequate reliability in the generation resources, to reduce costs and environmental pollution from energy production. This was where the artificial bee colony (ABC) algorithm offers a solution, as it had been used to optimize costs and minimize environmental pollution by finding the optimal production power of distributed generation.

The Archimedes Optimization Algorithm (AOA) was crafted by Al-Gazzar, M. M. et al. [27] drawing inspiration from the buoyancy principle and embodying a meta-heuristic optimization algorithm. The purpose of this algorithm was to identify the most cost-effective operation of interconnected microgrids (IMGs), which incorporate various forms of distributed generation (DG), namely solar photovoltaic (PV), wind turbine (WT), and micro-turbine (MT). The ultimate aim of this program was to minimize the aggregate cost of power generation, taking into account the exchange of power between the IMGs and the utility, all while factoring in any underlying technical constraints. The AOA algorithm's efficiency was manifested through a comparison with the particle swarm optimization algorithm (PSO), which represents another optimization method. The results of this comparison highlighted the potential of the AOA approach to curtail electricity consumption, decrease electricity costs and utility bills, and advance micro turbine (MT) performance for different daily loads by governing energy transfer between microgrids and the utility.

A metaheuristic algorithm was devised by Abaeifar, A. et al. [28] to tackle the EMS problem - the Inertia-Weight Local-Search based Teaching-Learning-Based Optimization (IWLS-TLBO). This approach was inspired by the human ability to learn, where self-perception and regulation are taken into consideration while making decisions based on past experiences. To assess the efficacy of the IWLS-TLBO algorithm with its predecessor, TLBO, and other metaheuristics, a comparison was drawn between the outcomes of the IWLS-TLBO algorithm on a range of benchmark functions and those yielded by TLBO and its counterparts. This analysis aimed to demonstrate the IWLS-TLBO algorithm's capacity to delve into uncharted territory and make the most of established solutions, thereby enabling more fruitful exploration of the search space. In the realm of isolated microgrids reliant on renewable energy sources, a quandary concerning the efficient allocation of resources plagued emergency management services. However, this issue was rectified through the optimization of a unit commitment problem via the IWLS-TLBO algorithm. The fruits of this labor were evidenced in simulation results that established the superiority of the IWLS-TLBO algorithm over other metaheuristic algorithms in both solution quality and convergence speed.

Reza Sepehrzad et al. [29] suggested a control strategy incorporating particle swarm optimization (PSO) and energy management algorithms to enhance the reliability, control levels, and incorporation of microgrids into existing electrical grids through improved power distribution. The proposed operational strategy relied on the load profile and power generation resources to predict power. To refine the energy management strategies, a multi-objective problem was solved with the PSO algorithm, and the optimization results were given to the fuzzy controller and power distribution management (PDM) unit. This improved power distribution in electrical grids. A comprehensive operating procedure for islanded and grid-connected microgrids, which takes into account their stability against grid fluctuations, was composed of an optimizer, a PDM unit, and a fuzzy controller. Additionally, to support the HESS and bolster its reliable performance, an auxiliary power control unit (APCU) is proposed. The utilization of MATLAB/Simulink was used to evaluate the effectiveness of the proposed structure when applied to the net power of islanded and grid-connected microgrids. This structure divides power into two components-high-frequency (super-capacitor) and low-frequency (battery and APCU). The results of the proposed algorithm and simulation were then analyzed.

The effectiveness of CSOS for solving the EMO problem in Microgrids was assessed by Omar, B. et al. [30] by using a chaotic symbiotic organism search (CSOS) algorithm. With the aid of a chaotic map, the approach was able to rapidly converge with a wider search space coverage when looking for solutions under various exploiting constraints. These results were then compared to those acquired from other scalable algorithms, such as GA and PSO, in terms of operating costs on a practical microgrid linked to public services. The comparison proved the efficiency of CSOS.

In terms of the objective function, the novelty of MCGA is not in the objective function itself but in the optimization method and techniques applied to the TEMS problem. The objective is to optimize the parameters and minimize the daily electricity price in an integrated clean energy microgrid. The contribution of the proposed approach is to modify and adapt the Chaos Grasshopper algorithm, specifically designed for TEMS in microgrids. The MCGA incorporates innovative strategies to enhance the optimization process and improve the solution quality. These modifications result in a significantly improved optimal solution for the overall daily electricity price compared to existing research approaches. By conducting comparative simulations with established methods like the Hybrid Optimization Model for Electric Renewables (HOMER), GAMS, Grey wolf optimizer (GWO), and Mixed-Integer Linear Programming Approach (MILPA), the effectiveness and superiority of the MCGA in achieving better solutions for the objective function are demonstrated. Therefore, while the objective function remains the same as in existing research, the novelty of the proposed approach lies in the modified Chaos Grasshopper Algorithm optimization, leading to improved results and contributing to the advancement of techno-economic energy management in microgrids.

Future scope: Future research should integrate emerging energy storage technologies, optimize power generation schedules, consider demand response mechanisms, evaluate advanced control strategies, and analyze environmental and economic factors. This will improve energy management and system efficiency, synchronizing energy supply and demand. Evaluating advanced control strategies' impact on performance, stability, and energy production can contribute to effective hybrid system management.

Advancements: The research study optimizes hybrid energy systems for efficiency, cost reduction, grid resilience, scalability, adaptability, and transferability. The algorithm intelligently manages energy sources, storage systems, and control mechanisms, resulting in increased energy production and reduced losses. Cost reduction is achieved by maximizing renewable energy production, minimizing fossil fuel-based power generation, and optimizing energy storage systems for peak demand periods. The algorithm's flexibility enhances grid resilience and can be applied to other renewable energy systems, improving efficiency and cost-effectiveness across multiple technologies.

Application: Optimizing hybrid energy systems can lead to various applications, such as renewable energy integration, microgrid and off-grid systems, energy cost savings, grid resilience, peak shaving, demand response, and decentralized power generation. These systems maximize renewable energy utilization, reduce fossil fuel dependence, and promote sustainable, efficient energy solutions, contributing to a cleaner and more resilient future.

Enhancements: The enhancements study focuses on optimizing hybrid energy systems by analyzing efficiency, reliability, and energy output. Researchers propose improvements in renewable energy technologies, battery storage capabilities, and advanced control algorithms. They explore energy storage technologies like advanced battery, compressed air, and hydrogen storage, and address grid integration and interconnection for seamless operation. The study proposes innovative solutions to improve performance, reliability, cost-effectiveness, and scalability, paving the way for a more sustainable and resilient energy infrastructure.

Constraints: Hybrid energy system optimization faces constraints such as technological limitations, resource availability, economic considerations, policy frameworks, stakeholder engagement, and integration. Technological limitations and site-specific factors impact performance and feasibility. Economic constraints, such as initial investment costs and payback periods, require addressing. Researchers can invest in renewable energy and energy storage technologies, and collaborate with policymakers and stakeholders to overcome these challenges.

## 2 System modeling

**Fig. 1:**
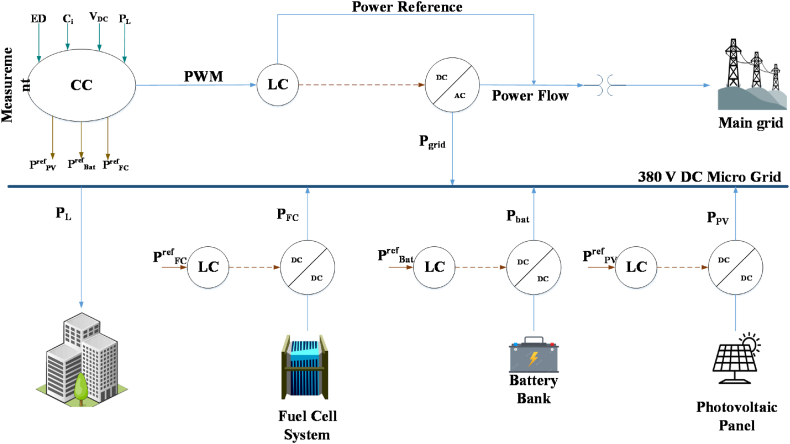
Structure of the proposed power system and its integrated components.

The primary objective of the current study is to investigate a microgrid that operates at low voltage and is connected to the main power grid. The microgrid is composed of various sub-systems, including a photovoltaic system (PV), a fuel cell (FC), a Lithium-ion battery energy storage system (BESS), and a range of loads present in the building. Fig. (1) illustrates the structure of the proposed power system and the different components that are integrated into it.

The exchange of electricity between the microgrid and the main power grid is carried out with the help of a transformer and a DC/AC converter. These devices are critical in ensuring a smooth and efficient flow of power between the two grids. The transformer serves to step down the high-voltage power from the main grid to a lower voltage that is suitable for the microgrid, while the DC/AC converter enables the conversion of DC power generated by the microgrid into AC power that can be utilized by the loads in the building.

To regulate the power generated by both the fuel cell and photovoltaic systems, a boost converter is employed. The boost converter steps up the voltage of the DC power generated by these systems to a level that is appropriate for use in the microgrid. This enables the microgrid to operate efficiently and ensures that the power generated by these systems is effectively utilized.

On the other hand, the power from the battery is managed using a buck/boost converter. This converter is used to regulate the voltage of the battery's DC power, enabling it to be efficiently integrated into the microgrid. The converter ensures that the power from the battery is utilized effectively and that any excess power generated by the microgrid is stored in the battery for future use.

Consequently, the integration of these various sub-systems and converters into the microgrid enables it to operate more efficiently, providing reliable and sustainable power to the loads present in the building. The current study aims to explore the performance of this microgrid in various operating conditions and to identify opportunities for further improvements. In the following, the mathematical modeling of the system components has been explained.

### 2.1 Mathematical model of the PV system

Solar cells are semiconductor devices that use the photovoltaic effect to turn sunlight into electrical energy. This technology has transformed power generation by providing a renewable and sustainable alternative to traditional energy sources like oil, gas, and coal.

When solar cells are joined together, they form a solar panel or module, which may generate electricity for a variety of uses, including powering homes and businesses and delivering energy to rural places that do not have access to the power grid. The mathematical model for the total power generated by the solar panel, which is a function of solar radiation and atmospheric conditions, is expressed as:

(1) $$P_{PV}=N_{pv}\times \left(P_{PV}^{POR}\times L_{PV}\times \frac{G_{h}^{t}}{G_{s}}\right)\times \left(1+\beta_{TP}\times \left(T_{C}-\bar{T}\right)\right)$$

where, $L_{PV}$ describes the solar panel loss factor due to the dirt, shadow, and temp, $\beta_{TP}$ specifies the power temperature coefficient, $P_{PV}^{POR}$ signifies the output power ratio capacity for the PV panel, $N_{\text{pv}}$ determines the PV panel ratio, $\bar{T}$ describes the PV temperature under standard test conditions, $G_{s}$ and $G_{h}\left(t\right)$ represent, in turn, the standard incident radiation ($1000W/m^{2}$), and the hourly PV panel solar radiation incident. $T_{c}$ here, is the solar cell temperature that is obtained as follows:

(2) $$T_{C}=T_{A}+\frac{NOCT-20{}^{\circ}}{0.8}\times G_{s}\left(\frac{\tau \times \alpha }{U_{l}}\right)$$

where, $T_{A}$ specifies the ambient temperature.

### 2.2 Fuel cell (FC) system

Eq. (3) has been commonly used to evaluate the performance of a single Solid Oxide Fuel Cell (SOFC) [4]. This equation enables us to quantitatively measure the voltage output of a SOFC based on its oxygen partial pressure, hydrogen partial pressure, and operating temperature. By using this Equation, we can effectively analyze the output voltage of a SOFC and assess its efficiency.

(3) $$E_{FC}=E_{N}-V_{act}-V_{\Omega }-V_{conc}$$

where, $V_{act}$ describes the activation voltage loss, $V_{\Omega }$ stands for the ohmic voltage loss, and $V_{cons}$ determines the concentration voltage loss. Here, the term $E_{N}$ specifies the Nernst reversible voltage and is achieved by the following equation:

(4) $$E_{N}=E_{0}+\frac{R\times T}{4\times F}\ln\left(\frac{{P_{O_{2}}\times P}_{H_{2}}^{2}}{P_{H_{2}O}^{2}}\right)$$

where, R specifies the gas universal constant equal to $8.314\;kJ\;{\left(kmol\;K\right)}^{-1}$, T signifies the fuel cell's operating temperature, F determines the Faraday constant which equals $96,486\;C\;mol^{-1}$, the standard potential is defined by $E_{0}$, and $P_{H_{2}}$, $P_{O_{2}}$, and $P_{H_{2}O}$ describe, in turn, the hydrogen, oxygen, and water partial pressures.

The concentration voltage loss of the fuel cell can be mathematically defined by the following equation:

(5) $$V_{conc}=\frac{R\times T}{4\times F}\times \left[\ln\left(\frac{P_{H_{2}}^{2}\times P_{O_{2}}}{P_{H_{2}O}^{2}}\right)-\ln\left(\frac{P_{H_{2}}^{*2}\times P_{O_{2}}^{*}}{P_{H_{2}O}^{*2}}\right)\right]$$

The ohmic voltage loss and the activation voltage loss can be achieved by the following equations:

(6) $$V_{\Omega }=I\times R_{\Omega }$$

(7) $$V_{act}=2\times \frac{R\times T}{n_{e}\times F}\times \sinh^{-1}\left(\frac{I}{{2\times I}_{0}}\right)$$

where, the gradually decreasing ionic resistance that relates to temperature increase, is defined by the term $R_{\Omega }$ [31]. Then, the current density of the element can be achieved by the following equation based on ButlereVolmer [32]:

(8) $$I=I_{0}\times \left[\exp\left(\frac{\tau \times n_{e}\times F\times V_{act,cell}}{R\times T}\right)-\exp\left(\frac{\left(\beta -1\right)\times n_{e}\times F\times V_{act,cell}}{R\times T}\right)\right]$$

Here, τ, $n_{e}$, and $I_{0}$ represent, in turn, the transfer factor, the moved electron moles, and the exchange current density. This paper assumes that τ=0.5 [33].

Finally, the main output voltage of the fuel cell can be achieved by the following equation:

(9) $$V_{FC}=N_{cell}{\times E}_{FC}$$

where, $N_{cell}$ signifies the number of combined cells.

And the output power of this element can be achieved by the following equation:

(10) $$P_{PV}=V_{FC}\times I\times A$$

where, A describes the available area.

### 2.3 Lithium-ion BESS

Due to its high energy density, extended lifespan, and quick charging capabilities, lithium-ion batteries are a preferred choice for microgrid and hybrid systems. They give the system a dependable source of energy storage, guaranteeing a constant flow of electricity independent of the weather or other environmental factors. As a result, they may be used in standalone, hybrid, and microgrid systems. They offer a cost-effective energy storage alternative that may assist lower power bills and achieve renewable energy targets. They are also perfect for peak flake and demand response applications.

A mathematical model of $N_{b}$ battery cells are formulated using two components: a voltage source ($V_{b}$) and internal resistance ($R_{b}$). The voltage equation for a battery cell incorporates parameters such as charge state and temperature.

(11) $$V_{B}=R_{Bat}\times I_{Bat}\pm N_{Bat}\times E_{Bat}$$

where, $I_{B}$ specifies the battery current, $E_{B}$ describes the potential of the battery, $N_{B}$ determines the lithium quantity, and $R_{B}$ determines the real part of the resistance $Z_{B}$, and is achieved as follows:

(12) $$R_{B}=\left|Z_{Bat}\right|\times \cos\;\varphi_{Bat}$$

where, $\varphi_{Bat}$ represents the phase shift.

### 2.4 System output

The relationship between the DC bus accessible energy ($E_{B}$) and the transferred power may be represented as a function, as depicted in Fig. (1).

(13) $${\dot{E}}_{B}=P_{G}+P_{PV}+P_{bat}-P_{L}$$

where, $P_{G}$, $P_{bat}$, $P_{L}$, and $P_{PV}$ represent, in turn, the generated energy for the grid, photovoltaic, battery, and load. Similarly, the DC energy bus has been achieved as follows:

(14) $$E_{B}=\frac{1}{2}\times C_{B}\times V_{B}^{2}$$

where $C_{B}$ and $V_{B}$ describe, in turn, the capacity and voltage bus.

A strategy for the optimal operation of a microgrid is being implemented on the central controller, which will consider load power, source operating costs ($C_{B}$), and electricity pricing ($E_{P}$). Additionally, this energy management system must also ensure adequate stability and power quality.

## 3 Fitness function

The photovoltaic power generated was insufficient to meet the required power, which resulted in a combination of power sources, including grid power, fuel cell, and battery, as determined by the chosen energy management strategy. The microgrid switched to grid mode for financial benefits, with grid power being the primary source of power unless the load exceeded the microgrid's power resources or the operating costs of these resources were excessive. This situation could be viewed as an optimization problem, where the aim is to minimize operating costs. The fitness function of the optimization problem can be represented by the following equation:

(15) $$Cost=\min\left(\sum_{t=1}^{T}\sum_{i=1}^{N}\left(C_{i}(P_{Gi}\left(t\right)+P_{Grid}\left(t\right)\times EP\left(t\right)\Delta T\right)\right)=\min\left(\sum_{t=1}^{T}\left(C_{FC}(P_{FC}\left(t\right)+C_{bat}\left(P_{bat}\left(t\right)\right)+P_{Grid}\left(t\right)\times EP\left(t\right)\Delta T\right)\right)$$

where, T and ΔT represent, in turn, the illustrates the total period, and the sample time, N the quantity of the power sources, EP(t) represents the pricing of the electricity market, $C_{i}$, $C_{bat}$, and $C_{FC}$ specify, in turn, the cost value of the $i^{th}$ DG, battery operation, and the fuel cell.

In comparison with existing research, the proposed approach introduces a new objective function in the optimization problem. The objective function in equation (15) represents the minimization of the overall cost of the microgrid's electricity consumption over a defined time period. The novelty of this objective function lies in its comprehensive consideration of multiple cost factors associated with different energy sources and grid electricity. Specifically, the objective function accounts for the costs related to fuel cell ($C_{FC}$), battery storage ($C_{bat}$), and grid electricity ($P_{Grid}\times EP\left(t\right)\Delta T$). By including these cost components, the proposed approach offers a more holistic perspective on the optimization problem. Previous research often focused on individual cost factors, such as minimizing the use of grid electricity or optimizing the operation of specific energy sources. In contrast, our proposed approach takes into account the combined impact of various cost factors, allowing for a more accurate representation of the real-world operational costs of a microgrid. This novel objective function enables the optimization algorithm to identify an optimal combination of energy generation and utilization strategies that minimize the overall cost. By considering multiple cost elements simultaneously, our approach offers a more comprehensive and realistic solution to the energy management problem in microgrids.

The function had several restrictions, such as power capacity and power equilibrium. To ensure power balance, the total power supplied by the resources must be equal to the load power for every time interval t, assuming no microgrid losses. Therefore, the power equilibrium constraint can be expressed by the following:

(16) $$P_{L}=P_{PV}+P_{bat}+P_{FC}+P_{G}$$

Moreover, power has to be generated within a certain capacity by the production units which possess their production means:

(17) $$P_{G}\in \left[P_{G}^{\min},P_{G}^{\max}\right]$$

Eq. (18) represents the operational cost of each generator as a quadratic equation with a single variable.

(18) $$C_{i}={\left(a_{i}P_{Gi}\right)}^{2}+b_{i}P_{Gi}+c_{i}$$

where, $a_{i}$, $b_{i}$, and $c_{i}$ define the cost coefficients.

The fitness function, therefore, places constraints on the output of the fuel cell and battery for power producers, which are as follows:

(19) $$\left[\begin{array}{c} P_{FC}^{\min}\\ P_{bat}^{\min} \end{array}\right]\leq \left[\begin{array}{c} P_{FC}\\ P_{bat} \end{array}\right]\leq \left[\begin{array}{c} P_{FC}^{\max}\\ P_{bat}^{\max} \end{array}\right]$$

(20) $$P_{G}=P_{L}-P_{FC}-P_{Bat}$$

This research studied how the state of charge of the battery can be integrated into the energy management strategy (EMS) to prevent severe drain or overcharge of the battery. A proposed fitness function was used to evaluate the battery's state of charge. This fitness function takes into account the power reference set forth by the energy management strategy, as well as microgrid sharing of the battery energy. It is important to note that some prior studies concerning an economic EMS did not include the battery state of charge in their analyses.

(21) $$F_{Cost}=\min\left(\sum_{t=1}^{T}\left(C_{FC}(P_{FC}\left(t\right)+C_{bat}.P_{bat}\left(t\right){\left(SoC\left(t\right)-SoC^{*}\left(t\right)\right)}^{2}+P_{G}\left(t\right)\times EP\left(t\right)\Delta T\right)\right)$$

where, $SoC^{*}\left(t\right)$ specifies the optimal rate of the SOC.

In this study, the optimization variables were the generator power references (including core grid power reference). To ensure power balance, the main grid power reference was not used as an optimization parameter. A revised version of the Grasshopper optimization algorithm has been proposed in this research to solve this problem. The photovoltaic cells, main grid power, and fuel cell power were employed to meet the demands, with the principal purpose of the battery being to maintain constant bus DC voltage. The flatness control approach can be used to stabilize the voltage and equation (22) can be utilized to determine the battery power reference:

(22) $${\hat{P}}_{bat}={\dot{E}}_{B}^{r}+P_{L}-\left(P_{G}+P_{PV}+P_{FC}\right)$$

where, ${\dot{E}}_{B}^{r}$ represents the required power of the common bus and is here obtained by the 2nd-order trajectory generation equation as follows:

(23) $$\frac{d\left(E_{B}-E_{B}^{r}\right)}{dt}+k_{1}\left(E_{B}-E_{B}^{r}\right)+k_{2}\int_{0}^{t}\left(E_{B}-E_{B}^{r}\right)dt=0$$

where, the $k_{1}$ and $k_{2}$ represent the trajectory generation coefficients and are attained as follows:

(24) $$k_{1}=2\zeta \omega_{n}$$

(25) $${k_{2}=\omega }_{n}^{2}$$

where, $\omega_{n}$ and ζ specify the cutoff frequency and damping ratio, and ζ=0.71.

## 4 Modified chaos grasshopper optimization algorithm

### 4.1 The standard grasshopper optimization algorithm

The grasshopper optimization algorithm (GOA) is new that is based on the group optimization process and that is inspired by the grasshopper insect's manner.

The individual contains a collective of grasshoppers which is named a group in this process. every organ of the swarm is a probable solution to the problem. The initial stage is commenced by producing a random group that is the primary problem's explanation. Then, every grasshopper's value is specified by gaining the objective value's cost.

The procedure is ongoing with attacking the group via selected grasshoppers into their place to imbibe the grasshoppers to transfer into the selected grasshopper.

In this present study, the grasshoppers' 2 major manners are studied: the larval grasshoppers’ sluggish and ticonsiderny motion with the aim of the long-range and no subsequence motion move matures, also nourishment probing procedure which is separated into 2 distinct portions of use and search.

$Z_{i\;}$ describes the $i^{th}$ grasshopper's improvement away from the objective grasshopper that is calculated by the next Equation:

(26) $$Z_{i}=b_{1}S_{i}+{b_{2}G}_{i}+b_{3}W_{i}$$

(27) $$P_{i}=R_{1}SA_{i}+R_{2}GF_{i}+R_{3}WA_{i}$$

where, $W_{i\;}$, $S_{i\;}$ and $G_{i\;}$ define the wind advection, the social contact, and the gravity force on the ith grasshopper, respectively. $b_{1}$, $b_{2}$, and $b_{3\;}$ are random coefficients that are between 0 and 1.

The $i^{th}$ grasshopper social contact ($S_{i\;}$) is connected to the social forces that are amid 2 grasshoppers and is an excretion force to end encounters and an absorption force aim over a minor distance scale.

(28) $$S_{i}=\sum_{j=1\;}^{N}=S\left(P_{ij}\right){\hat{P}}_{ij}$$

(29) $$P_{ij}=Y_{i}-Y_{k}$$

(30) $${\hat{P}}_{ij}=\frac{Y_{i}-Y_{k}}{P_{ij}}$$

where, $P_{ij}$ determines the Euclidian's length of the *i*th with the *j*th place grasshopper, and $P_{ij}$ signifies the current unit vector among the *i*th grasshopper and the *j*th grasshopper.

The next equation illustrates the severity absorption power:

(31) $$Y_{i}^{d}=c\left(\sum_{\begin{array}{c} j=1\\ j\neq i \end{array}}^{N}c\frac{u_{b}^{d}-L_{b}^{d}}{2}S\left(\left|Y_{j}^{d}-Y_{i}^{d}\right|\right)\frac{Y_{j}-Y_{i}}{P_{ij}}\right)+{\hat{T}}_{d}$$

(32) $$C=c_{\max}-l\frac{c_{\max}-c_{\min}}{L}$$

where N is the quantity of grasshopper, $u_{b}^{d}$ and $L_{b}^{d}$ are upper and the lower restrictions in the dth dimension, respectively, $\overset{\wedge }{T_{d}}$ does aim measure of dth dimension by the aimed grasshopper, c designates the decreasing element to the suitable area of the excretion and absorption region, $c_{\max}$ and $c_{\min}$ are the max and the min cost of c, respectively, and finally, l and L are the present repetitions and the repetition's complete number, respectively.

Where S indicates the social forces’ strong points that are estimated by the next formula:

(33) $$s\left(b_{ij}\right)=f_{i}e^{\frac{-b}{1}}-e^{b}$$

where, l determines the absorption's length scale, and fi is the severity force of absorption power.

G is the process's parameter that is obtained as follows:

(34) $$G_{i}=-G.{\hat{e}}_{g}$$

where, *G*_*i*_ indicates a constant factor for the gravity force, and a direction for the unity vector beside the wind is described by ${\hat{e}}_{g}$A1

finally, the wind advection (*W*_*i*_) is gained as follows:

(35) $$W_{i}=-u.{\hat{e}}_{g}$$

where the drift constant factor is determined by *u*.

### 4.2 Modified chaos grasshopper optimization algorithm

For enhancing the speed of convergence in this method, a method is planned for adapting the GAO's crucial parameters. The GAO convergence's vital parameters are *u, b*_*1*_*, b*_*2*_*, and b*_*3*_ the adjustment is applied on the basis of chaos theory.

Chaos theory is the research of changeable and uncertain procedures. The chaos theory's central idea is to study the very susceptible dynamic organisms that any tiny variants could influence it. To the explanations that are mentioned above, a major variety could be created for the group generation in GOA to enhance the algorithm's variety. This portion could enhance the GOA algorithm's ability to the speed of convergence and for evasion from dropping to the local optimal point [34,35].(Yang et al., 2007, Rim et al., 2018)(Yang et al., 2007, Rim et al., 2018)(Yang et al., 2007, Rim et al., 2018)(Yang et al., 2007, Rim et al., 2018)(Yang et al., 2007, Rim et al., 2018) [10,10,10,10,11,11,11,11]A common formula for the chaos theory is shown as follows:

(36) $${CM}_{i+1}^{j}=f\left({CM}_{i}^{j}\right)\;j=1,2,\ldots ,k$$

where the dimension of the map is demonstrated by *k*, and the disordered plan producer function is described by $f\left({CM}_{i}^{j}\right)$.

In the existing GOA, the parameters *u*, *b*_*1*_*, b*_*2*_*, and b*_*3*_ are formulated based on the Kent map as the next equation:

(37) $$b_{i+1}=\left\{ \begin{array}{c} \frac{b_{i}}{\theta },0<b_{i}\leq \theta \\ \frac{\left(1-b_{i}\right)}{\left(1-\theta \right)},\theta <b_{i}\leq 1 \end{array}\right.$$

(38) $$u_{i+1}=\left\{ \begin{array}{c} \frac{u_{i}}{\theta },0<b_{i}\leq \theta \\ \frac{\left(1-u_{i}\right)}{\left(1-\theta \right)},\theta <b_{i}\leq 1 \end{array}\right.$$

where, θ is a variable between 0 and 1. Here, θ=0.5A1

**Fig. 2:**
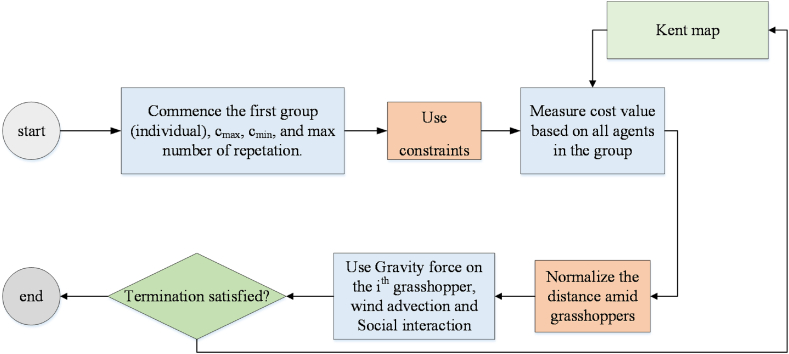
The offered MCGA's flowchart.

Fig. (2) illustrates the offered CGOA's flowchart.

To further demonstrate the efficacy of the proposed MCGA, four unimodal and multimodal test functions were employed, and the results were compared with those yielded by certain benchmark algorithms, such as Pigeon-inspired Optimization Algorithm (PIO) [36], Squirrel search algorithm (SSA) [37], Spotted hyena optimize (SHO) [38], Lion optimization algorithm (LOA) [39], and basic GOA [40]. The detailed parameter settings used for all optimized algorithms are shown in Table 1.

**Table 1:** Detailed parameter settings used for all optimized algorithms.

Algorithm	Parameter name	Value
Pigeon-inspired Optimization Algorithm (PIO) [36]	Number of Pigeons	120
	Space dimension	15
	Map and compass factor	0.3
	Map and compass operation limit	100
	Landmark operation limit	120
	Inertia factor (w)	2
	Self-confidence factor ($c_{1}$)	1.5
	Swarm confidence factor ($c_{2}$)	1.5
Squirrel search algorithm (SSA) [37]	$N_{fs}$	5
	$G_{c}$	2
	$P_{dp}$	0.3
Spotted hyena optimization (SHO) [38]	$\vec{M}$	0.5
	$\vec{h}$	3
Lion optimization algorithm (LOA) [39]	Number of prides	4
	Percent of nomad lions	0.2
	Roaming percent	0.3
	Mutate probability	0.2
	Sex rate	0.9
	Mating probability	0.5
	Immigrate rate	0.4

In many cases, benchmarks are used to evaluate the performance of algorithms and their ability to solve specific problems. As such, the benchmarks listed in Table 1 likely played an essential role in demonstrating the effectiveness and practicality of the algorithms being evaluated. Table 2 provides a comprehensive summary of the benchmarks that were used for validating various algorithms. It includes a range of details related to these benchmarks, such as their names, mathematical formulation, and constraint. Accurate and comprehensive validation is critical to establishing the credibility and usefulness of any algorithm, and Table 2 provides important information about the benchmarks used to support this validation process.

**Table 2:** The information of test functions adopted for algorithm verification.

Tests function	Equation	Limitation
Rastrigin	$f_{1}\left(x\right)=10D+\sum_{i=1}^{D}\left(x_{i}^{2}-10\;\cos\left(2\pi x_{i}\right)\right)$	[-608, 608]
Rosenbrock	$f_{2}\left(x\right)=\sum_{i=1}^{D-1}\left(100\left(x_{i}^{2}-x_{i+1}\right)+{\left(x_{i}-1\right)}^{2}\right)$	[-2.982, 2.982]
Ackley	$f_{3}\left(x\right)=20+e-20\;\exp\left(-0.2\sqrt{\frac{1}{D}\sum_{i=1}^{D}\left(x_{i}^{2}\right)}\right)-\exp\left(\frac{1}{D}\sum_{i=1}^{D}\left(\cos\left(2\pi x_{i}\right)\right)\right)$	[-11.50, 11.50]
Sphere	$f_{4}\left(x\right)=\sum_{i=1}^{D}x_{i}^{2}$	[-608, 608]

Table 3 indicates the validation of the MCOA algorithm compared to the other analyzed approaches. The results in Table 3 show that, among all the algorithms, MCGA has achieved the lowest minimum value and the highest median value, indicating that it is the most effective algorithm for this problem. In addition, MCGA has a lower standard deviation value than other algorithms, which further confirms its robustness and efficacy. Moreover, DE has achieved a better maximum value than other algorithms, but its median value was not as good as the others, suggesting that the performance of DE was not consistent across runs.

**Table 3:** Validation of the MCGA algorithm compared to the other analyzed approaches.

Benchmark			MCGA	GOA [40]	PIO [36]	SSA [37]	SHO [38]	LOA [39]
F1	AVE	Value	0.02	2.47	3.28	3.82	4.22	4.53
		Rank	2	4	7	9	11	15
	STD	Value	0.02	2.52	3.72	3.60	3.25	4.53
		Rank	3	4	7	9	10	12
	Friedman test	Value	3.14	3.25	3.64	4.05	4.26	4.53
		Rank	4	6	8	9	10	12
F2	AVE	Value	0.58	1.16	1.24	1.34	2.52	2.75
		Rank	6	9	11	15	18	21
	STD	Value	0.77	0.81	0.95	1.03	1.17	1.54
		Rank	10	13	15	17	20	22
	Friedman test	Value	2.19	2.51	2.86	3.14	3.46	3.81
		Rank	11	15	18	22	25	28
F3	AVE	Value	4.14e-12	5.18e-11	4.24e-10	2.32e-8	2.43e-8	2.354e-7
		Rank	12	16	20	23	26	28
	STD	Value	7.15e-12	8.20e-11	7.53e-10	3.47e-8	3.13e-8	4.33e-7
		Rank	13	18	21	25	26	29
	Friedman test	Value	4.25	4.76	5.60	5.49	5.63	6.22
		Rank	15	20	23	25	9	31
F4	AVE	Value	0.00	0.00	8.95e-10	0.00	3.52e-9	3.11e-8
		Rank	5	8	10	11	12	15
	STD	Value	0.00	0.00	8.22e-10	0.00	3.53e-9	3.22e-8
		Rank	7	9	12	15	18	21
	Friedman test	Value	4.32	4.63	4.84	5.43	5.62	5.71
		Rank	3	5	7	10	14	16

**Fig. 3:**
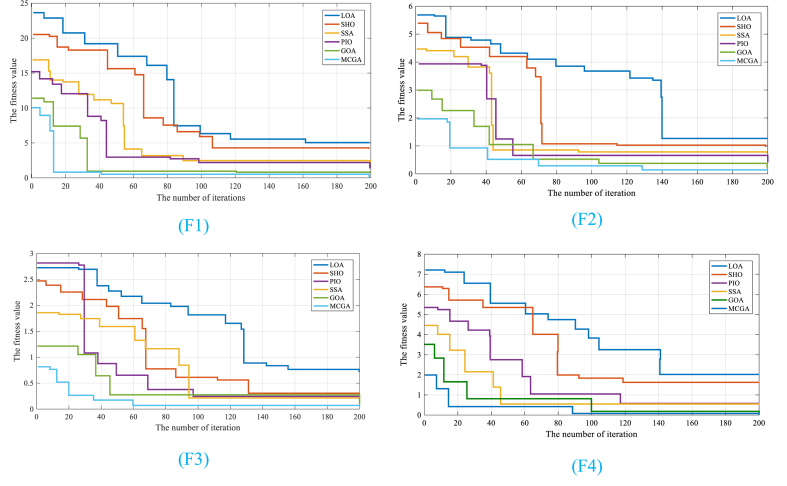
The algorithms' convergence results for function 1 (F1), function 2 (F2), function 3 (F3), function 4 (F4).

The convergence index is a commonly used metric to evaluate the performance of optimization algorithms. This index measures how quickly an algorithm can reach the global optimum solution of a given problem. The faster an algorithm converges to the global optimum, its performance improves. In optimization algorithms, individuals represent candidate solutions evaluated and modified iteratively in search of the optimal solution. During the initial phase of the optimization process, individuals explore the entire search space quickly to identify potential solutions. As the optimization continues, individuals are refined, and the search space is narrowed to the most promising regions. Fig. (3) shows the results of the convergence of different algorithms on standard functions. The convergence curve represents the rate at which the algorithm converges to the global optimum solution.

The improved MCGA exhibits superior performance in convergence speed and exploration-exploitation balance compared to other optimization algorithms. Its goal is to prevent early convergence and promote rapid convergence, making it a promising solution for optimization problems. The convergence curve in Fig. (3) shows rapid convergence during initial iterations, indicating individuals exploring the search space at a faster rate. This allows the improved MCGA to identify potential solutions more efficiently and effectively, leading to faster convergence.

The results suggest that the MCGA algorithm outperformed other algorithms being compared to it, achieving the minimum value for all the analyzed test functions. This indicates that the algorithm was successful at finding the optimal solution or best possible output for each test function. Moreover, the statement highlights another key aspect of the algorithm's performance, the standard deviation. By comparing the standard deviation of MCGA with other algorithms, it was found that MCGA has the lowest standard deviation. In other words, the algorithm's output was relatively consistent across multiple runs, and the variation between different iterations was minimal. This is a positive indication as it signifies that the algorithm was able to converge to the optimal solution effectively without getting stuck in a suboptimal solution, which could lead to high variance.

## 5 Results and discussions

**Fig. 4:**
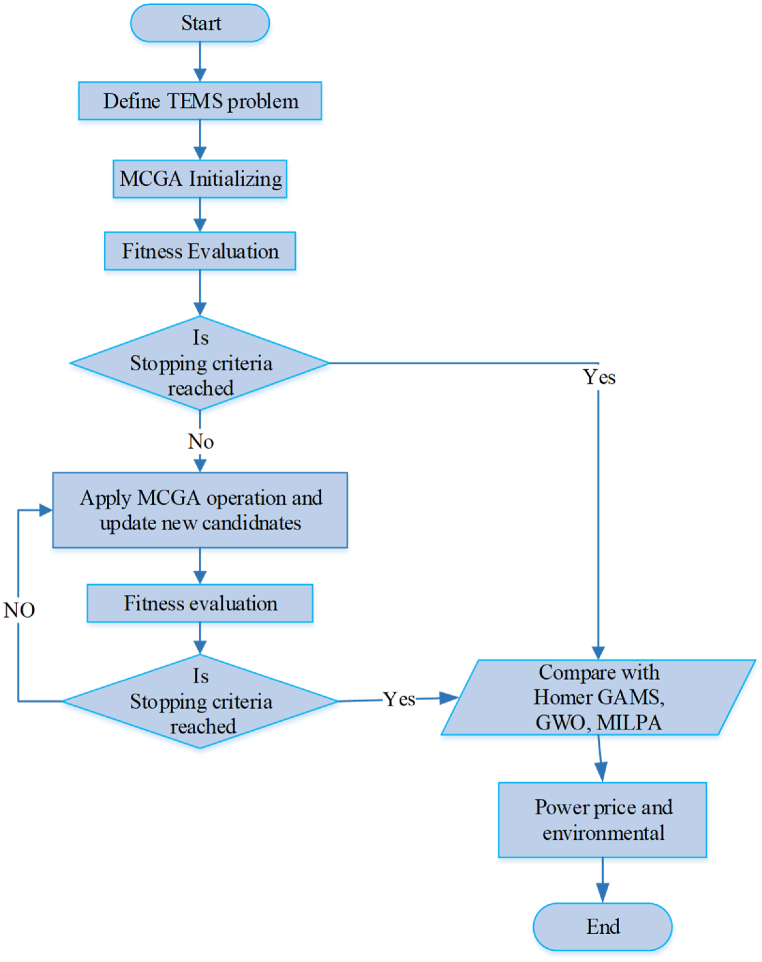
Flowchart diagram of the methodology steps.

This research aims to develop a new approach to optimize the energy production of a hybrid system. The proposed approach involves using an optimization algorithm to determine the optimal operating conditions of the hybrid system components, such as Photovoltaic, Fuel Cells, and BESS, to maximize energy production. Fig. (4) shows a flowchart of the methodology steps.

To ensure the efficient operation of the microgrid, it was necessary to simulate its performance under varying conditions. The simulation was carried out using solar radiance and load fluctuation as inputs to test the effectiveness of the energy management strategy that had been proposed. The micro-grid system that was used in the simulation had a DC bus voltage of 400 V and a maximum power output of 352.2 kW. It also had a battery capacity of 1400 Ah, with minimum and maximum values for the state of charge (SOC) set at 8 and 68% respectively.

**Fig. 5:**
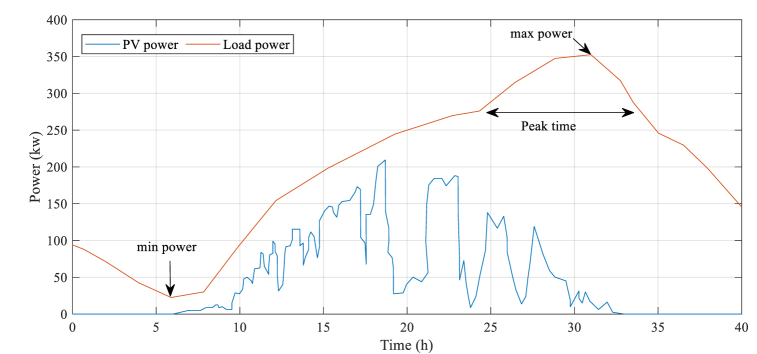
Typical power generated by PV and consumed by loads in the system under investigation.

Fig. (5) provides a visual representation of the typical PV power and load power profiles for the microgrid system that was studied. The graph shows how these two parameters vary over time, with PV power being generated during daylight hours and load power fluctuating throughout the day.

The simulation allowed for an in-depth analysis of how the energy management strategy would perform under different scenarios. By testing the system's response to varying levels of solar radiance and load fluctuations, it was possible to identify any potential issues or areas for improvement.

Consequently, the simulation provided valuable insights into how the microgrid system would operate in real-world conditions. By using this information, it is possible to optimize its performance and ensure that it meets the energy needs of those who rely on it. Fig. (5) illustrates the typical power generated by PV and consumed by loads in the system under investigation.

Cost parameters have been supplied for both the load and the PV systems (probably referring to solar installations) based on [41]. These cost parameters are probably connected to some modeling or simulation that is being done, potentially in the area of electrical engineering or renewable energy.

**Fig. 6:**
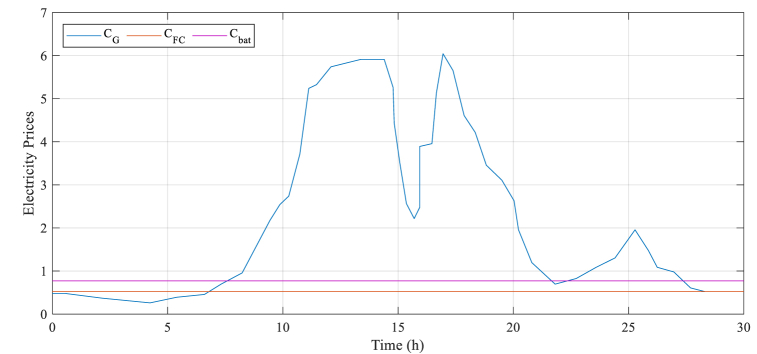
System electricity price.

Fig. (6) associated with [41] is being used to provide these cost parameters. This figure could potentially include a graph or chart displaying the different costs associated with implementing and operating the load and PV systems.

Understanding the economics of the system is an important case. The price of electricity can vary widely depending on where it is generated, how it is generated, and who is supplying it. Factors like government subsidies, infrastructure costs, and maintenance costs can all play a role in determining the final price that consumers pay for electricity. As the world shifts towards renewable energy sources, we can expect the cost of electricity generated by wind, solar, and other renewable technologies to continue to fall, making them more competitive with traditional power sources.

**Fig. 7:**
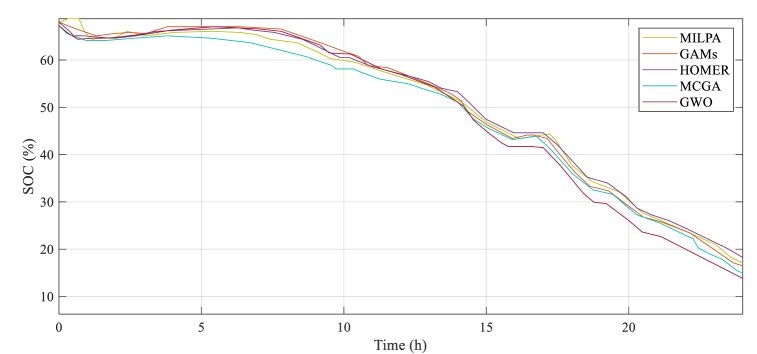
The battery state of charge (SoC) profile for various methods.

The Modified Chaos Grasshopper Algorithm was compared with different optimization algorithms such as Hybrid Optimization Model for Electric Renewables (HOMER) [42], GAMS [43], Grey wolf optimization (GWO) [20], Mixed-Integer Linear Programming Approach (MILPA) [44] to evaluate its efficiency. As illustrated in Fig. (6), the power demand was higher than the available solar power. To provide enough power, the energy management strategy proposed assigning a power reference for the grid, battery, and fuel cell. The optimal solution is shown in Fig. (7) and indicates that the suggested algorithm was successful.

Two principal electricity pricing periods—from 6:30 to 24:00 and from 24:00 to 6:30—are depicted in Fig. (7). Battery power and fuel cell energy are more expensive than grid electricity during the low grid pricing era. As a result of the lower power rates during the 1 to 6-h and 24-h periods, the main grid can provide the bulk of the demand. After six in the morning, the fuel cell takes over for energy production, and the battery supplies energy when the weather changes. After 14:00, grid prices started to decline, which led to an increase in load power that peaked at 16:00.

The fuel cell is usually the critical energy source during this timeframe, as evidenced by the electricity profiles. However, the power from the grid is regulated by market tariffs. The battery's state of charge is used to manage the exchanged power. Fig. (7) displays the battery SoC profiles for multiple comparison techniques. Table 4 includes data and simulation results for the MCGA and compares it with other methods that were studied. The table records essential metrics such as the final “SoC” (State of Charge), which refers to the amount of energy stored in a battery or cell, as well as the method's efficiency, which reflects the ratio of energy output to energy input. Additionally, the table includes information about optimizer efficiency, which is a measure of how well the method can find optimal solutions, and the mean operating cost, which indicates how expensive it is to operate using this method on average. By examining these values and comparing them to other methods, researchers can gain insights into the effectiveness and practicality of using the MCGA method for their purposes. So, the data presented in Table 4 is crucial for understanding the simulation results and making informed decisions based on them.

**Table 4:** The results of the simulation, including final SoC, efficiency, optimizer efficiency, and mean operating cost.

Run	GAMS		MILPA		GWO		HOMER		MCGA	
	Sys. Efficiency (%)	Final SOC (%)	Sys. Efficiency (%)	Final SOC (%)	Sys. Efficiency (%)	Final SOC (%)	Sys. Efficiency (%)	Final SOC (%)	Sys. Efficiency (%)	Final SOC (%)
1	86.12	31.53	83.23	28.43	84.25	4.69	85.69	32.16	87.68	33.32
2	86.25	31.52	83.29	28.42	84.22	4.57	85.68	32.13	87.85	33.28
3	85.72	31.29	83.24	28.52	84.21	4.42	85.68	32.25	87.85	33.29
4	86.16	31.42	83.32	28.65	84.22	3.79	85.76	32.23	87.73	33.27
5	86.28	31.42	83.18	28.49	84.19	4.49	85.68	32.18	87.79	33.36
6	86.26	31.39	83.29	28.47	84.18	4.53	85.83	32.29	87.79	33.39
7	86.26	30.25	82.22	28.31	84.36	4.16	85.72	32.17	87.77	33.41
8	86.21	31.42	83.13	28.67	84.33	3.77	85.66	32.15	87.83	33.34
9	86.27	31.28	83.32	28.79	84.22	3.68	85.64	32.14	87.86	33.33
10	85.82	31.43	83.27	28.49	84.23	4.36	85.54	32.22	87.93	33.39
Best	85.73	31.29	82.89	28.32	84.21	3.75	85.54	32.13	87.81	33.33
Worst	86.22	31.67	83.38	28.79	84.36	4.69	85.83	32.29	87.98	33.28
Mean	86.36	31.56	83.12	28.51	84.32	4.62	85.69	32.18	87.88	33.32
Median	86.42	31.53	83.12	28.66	84.33	4.75	85.69	32.21	87.91	33.29
StD	0.013	0.0124	0.025	0.015	0.0057	0.025	0.008	0.010	0.0038	0.0056
Run	Cost daily	Cost/power ($/kw)	Cost daily	Cost/power ($/kw)	Cost daily	Cost/power ($/kw)	Cost daily	Cost/power ($/kw)	Cost daily	Cost/power ($/kW)
1	1,368,355	0.1786	2,526,193	0.4261	3,212,432	0.3453	1.593,717	0.1978	1,294,916	0.1576
2	1,368,819	0.1789	2,521,798	0.4255	3,214,941	0.3455	1,594,695	0.1981	1,294,381	0.1577
3	1,368,467	0.1785	2,522,000	0.4255	3,213,218	0.3453	1,594,752	0.1981	1,294,523	0.1577
4	1,369,484	0.1787	2,523,270	0.4257	3,211,843	0.3452	1,595,671	0.1983	1,294,541	0.1577
5	1,369,455	0.1787	2,525,343	0.426	3,212,625	0.3453	1,595,156	0.1982	1,294,662	0.1577
6	1,368,433	0.1785	2,521,875	0.4255	3,211,561	0.3452	1,595,345	0.1982	1,294,988	0.1578
7	1,368,855	0.1786	2,523,328	0.4257	3,213,369	0.3454	1,595,925	0.1983	1,294,612	0.1577
8	1,369,636	0.1787	2,525,134	0.4259	3,214,231	0.3453	1,595,928	0.1983	1,294,818	0.1577
9	1,368,541	0.1785	2,521,179	0.4252	3,212,219	0.3453	1,592,751	0.1979	1,294,559	0.1577
10	1,368,898	0.15786	2,519,500	0.4252	3,212,144	0.3453	1,594,000	0.1981	1,294,916	0.1578
Best	1,368,355	0.1785	2,519,500	0.4252	3,211,843	0.3452	1,592,751	0.1979	1,294,381	0.158
Worst	1,369,636	0.1786	2,526,193	0.4261	3,214,941	0.3455	1,595,928	0.1983	1,294,988	0.1578
Mean	1,368,893	0.1786	2,522,843	0.4256	3,212,736	0.3453	1,593,785	0.1981	1,294,688	0.1577
Median	1,368,832	0.1786	2,422,641	0.4256	3,212,529	0.3453	1,594,914	0.1982	1,294,632	0.1577
StD	472.933	5.21^-4	2216657.4	30.21^-5	1213,793	8.25^-6	1032.363	13.21^-5	212.121	2.21^-5
Efficiency	93.54		48.53		64.28		79.58		98.97	

Table 4 in the study compares the battery's efficiency and final state of charge (SoC) using different metaheuristic algorithms. The results indicate that the proposed MCGA provides better results than the other comparative methods. However, the values obtained are low, which means there is still room for improvement in the performance of the proposed method. Therefore, the study concludes that using metaheuristic-based algorithms to optimize techno-economic energy management strategies in microgrids is useful. The proposed method provides a more effective optimal solution than the other current approaches, with high precision and flexibility, and resistance to changes in power prices and environmental constraints. In summary, the study demonstrates the potential of metaheuristic algorithms for optimizing the performance of techno-economic energy management strategies in microgrids. The proposed Modified Chaos Grasshopper Algorithm provides a more effective solution than other current approaches. However, the study also highlights the need for further improvements in the performance of the proposed method.

## 6 Conclusions

The present study proposes a modified Grasshopper Optimization Algorithm (MCGA) as a techno-economic energy management strategy (TEMS) optimization technique in microgrids. The method is tested on an integrated clean energy microgrid comprising a fuel cell, battery storage, and photovoltaic system in independent and grid-connected modes. To assess its effectiveness, the proposed strategy is compared with other optimization models such as HOMER, GAMS, GWO, and MILPA. The findings demonstrated the performance of different metaheuristic algorithms in optimizing the techno-economic energy management strategies in microgrids. The Modified Chaos Grasshopper Algorithm (MCGA) consistently outperformed other comparative methods, with a mean final state of charge (SoC) of 33.29 % and system efficiency of 87.91 %. In contrast, HOMER, GWO, MILPA, and GAMS achieved lower mean SoC values of 32.21 %, 4.75 %, 28.66 %, and 31.53 % respectively, along with lower system efficiencies ranging from 85.54 % to 85.69 %. Furthermore, MCGA exhibited the highest best-case and median SoC values of 33.28 % and 33.29 %, surpassing the other methods. The worst-case SoC value for MCGA was 33.32 %, indicating its robustness compared to the other algorithms. In terms of system efficiency, MCGA also demonstrated superiority, with the highest best-case and median values of 87.98 % and 87.91 % respectively. The worst-case efficiency for MCGA was 87.88 %. Examining the standard deviations (StD) of the cost per power values, MCGA achieved a relatively low StD of 0.0124$/kW, suggesting consistency in cost effectiveness. Similarly, MCGA exhibited a lower StD for daily cost compared to the other methods, indicating its stability in achieving cost-efficient operation. The simulation results demonstrate that the proposed approach effectively reduces the daily electricity price and optimizes the microgrid components' parameters. Compared to the alternative models, the suggested algorithm significantly improves precision, flexibility, and resistance to changes in power prices and environmental constraints. Therefore, the MCGA technique is a practical and effective solution for optimizing microgrids' techno-economic energy management strategy. Future research endeavors could explore the application of the MCGA technique in other renewable energy systems or larger-scale microgrid systems. Furthermore, this study highlights the importance of integrating renewable energy sources, implementing efficient control strategies, and adopting energy storage technologies in hybrid energy systems. While renewable sources maximize utilization and reduce reliance on fossil fuels, their intermittent nature poses certain constraints. Energy storage technologies offer enhanced capacity, longer storage duration, and improved system performance, yet high costs may impede widespread adoption. Ensuring grid integration and interconnection is crucial for scalable deployment, and cost-reduction strategies can enhance affordability. Finally, scalability and flexibility are essential for successfully implementing hybrid energy systems.

## CRediT authorship contribution statement

**Zhiyu Yan:** Writing - original draft, Formal analysis, Data curation. **Yimeng Li:** Writing - review & editing, Writing - original draft, Software, Conceptualization. **Mahdiyeh Eslami:** Writing - review & editing, Writing - original draft, Software, Resources, Formal analysis, Data curation.

## Declaration of competing interest

The authors declare that they have no known competing financial interests or personal relationships that could have appeared to influence the work reported in this paper.
